# Identification of ferroptosis-related gene signatures as a novel prognostic model for clear cell renal cell carcinoma

**DOI:** 10.1007/s12672-025-02202-1

**Published:** 2025-04-03

**Authors:** Xiaoxiao Du, Haoyuan Cao, Yu-Jie Zhou, Qingli Kong, Xulong Zhang

**Affiliations:** 1 Department of Immunology, School of Basic Medical Sciences, Capital Medical University, Beijing, 100069 China; 2 Department of Urology, Beijing Chao-Yang Hospital, Capital Medical University, Beijing, China; 3 HBV Infection, Clinical Cure and Immunology Joint Laboratory for Clinical Medicine, Capital Medical University, Beijing, China

**Keywords:** Ferroptosis, Prognostic model, CcRcc, Immune

## Abstract

**Background:**

Clear cell renal cell carcinoma (ccRCC), a common type of renal cortical tumor, is the most prevalent subtype of renal malignancies within the urinary system and is associated with a low survival rate. Ferroptosis plays a crucial role in the process of renal carcinogenesis and holds potential for significant applications in patient prognosis. However, the clinical prognostic relevance of ferroptosis-related genes (FRGs) for ccRCC remains unclear. The identification of FRG signatures and the development of a novel prognostic model based on FRGs demonstrate important prognostic significance for ccRCC.

**Methods:**

Univariate cox screen was performed to screen for prognostic-related genes using ccRCC data from the The Cancer Genome Atlas (TCGA) database. And then an initial screen for prognostic genes was performed by taking intersections with the differential genes of the Gene Expression Omnibus (GEO) database datasets GSE213324 and GSE66271, as well as with the FRGs, and a multigene signature was constructed using least absolute shrinkage and selection operator (LASSO) and Cox regression analysis. Subsequently, the model was evaluated using Kaplan–Meier (KM) survival curve analysis, receiver operating characteristic (ROC), nomogram, and decision curve analysis (DCA). Differences in tumor microenvironment and immune function were analyzed by single-sample gene set enrichment analysis (ssGSEA) and immune infiltration in patients in the high- and low-risk groups. The tumor immune dysfunction and exclusion (TIDE) assessed the immune checkpoint inhibitor (ICI) susceptibility in patients. The Gene Set Enrichment Analysis (GSEA) was performed for pathway enrichment analysis. Patient mutation data were downloaded and tumor mutation burden (TMB) were compared between patients in the high- and low-risk groups.

**Results:**

*ADACSB*, *DPEP1*, *KIF20A*, *MT1G*, *PVT1* and *TIMP1* were utilized to establish a novel prognostic signature. The KM curve analysis revealed that patients in the high-risk group exhibited a poorer prognosis. Additionally, the ROC results demonstrated that the model displayed favorable prognostic accuracy. Independent prognostic analyses indicated that the FRGs model could serve as an independent prognostic indicator. Furthermore, calibration curve of the nomogram illustrated enhanced precision in predicting survival rates for patients at 1, 3 and 5 years. Analysis of mutation data unveiled higher tumor mutation load among patients in the high-risk group, which correlated with an increase in risk score.

**Conclusion:**

The FRGs model offers a novel approach for prognostic prediction of ccRCC patients and has the potential to provide personalized prognostic prediction and treatment for ccRCC patients.

**Supplementary Information:**

The online version contains supplementary material available at 10.1007/s12672-025-02202-1.

## Introduction

Cancer-related human mortality remains a challenging issue on a global scale, with important implications for overall survival rates [1]. As the urological tumor, ccRCC stands out as the most common malignant tumor in renal cancer, and its incidence and mortality rates continue to rise [2]. According to the Global Cancer Statistics 2020, the number of new cases will be about 430,000, with 180,000 deaths [3]. Furthermore, ccRcc is characterized by high invasiveness and metastatic potential, while patients exhibit low resistance to radiotherapy and poor prognosis [4]. Therefore, more accurate prognostic indicators are needed for clinicians to diagnose and treat the disease, which will help patients to improve their survival rates and living conditions.

Programmed cell death (PCD) is crucial for physiological development, maintenance of homeostasis, and regulation of disease development [5]. Ferroptosis is an iron-dependent mode of PCD, resulting from the excessive accumulation of lipid peroxides due to disturbances in intracellular metabolic pathways, which is intimately related to intracellular iron metabolism and lipid homeostasis [6, 7] and distinguishes it from other forms of death such as apoptosis, necrosis, autophagy. In recent years, a growing body of evidence has demonstrated the association between ferroptosis and the development and advancement of malignant tumors including ccRCC, hepatocellular carcinoma (HCC), and colorectal cancer [8], underscoring its significance in tumor therapy. Consequently, searching for more ferroptosis-related biomarkers has a profound importance for the prognosis and treatment of patients with ccRcc. For example, cysteine protease inhibitor SN (CST1) could regulate the protein stability of glutathione peroxidase 4 (GPX4) through OTUB1 and inhibit the ferroptosis of gastric cancer cells, which in turn promoted the metastasis of gastric cancer to the liver, lungs and peritoneum [9]. High expression of CST1 was associated with a poor prognosis for patients [9]. Otherwise, many other studies have reported the outcome of ferroptosis and ccRCC. Augmented circular RNA zinc finger with KRAB and SCAN domains 1 (circZKSCAN1) expression facilitated ccRCC development and accelerated tumor progression by targeting the miR-1294/ Pim-1 proto-oncogene, serine/threonine kinase (PIM1) axis [10]. It has also been found that Krüppel-like factor 11(KLF11) suppressed the progression of ccRCC by increasing the transcription of nuclear receptor coactivator 4 (NCOA4), which may be a therapeutic target for ccRCC [11]. Moreover, a significant correlation between ferroptosis and ccRcc prognosis was found recently [12–14]. These findings suggested that ferroptosis plays important roles in the development and progression of ccRCC and is closely linked to the prognosis of ccRcc patients. However, with the limited available research in this area, the relationship between ccRCC prognosis and FRGs remains unclear. Therefore, identifying for ferroptosis-related markers in ccRCC may further enhance our understanding of the role of ferroptosis in ccRCC, as well as show very important roles in prognosis and treatment of patient.

Although the research has reported the association of FRGs with the prognosis of certain cancers, there is limited information on the clinical prognostic relevance of FRGs for ccRCC. In this study, a multi-gene prognostic model related to ferroptosis was screened and established based on data from TCGA and the ferroptosis database (FerrDb), which could independently predict the prognosis of ccRCC patients. These findings provide theoretical references for the prevention and treatment of ccRCC.

## Materials and methods

### Data collection and acquisition

RNA-seq data related to ccRCC were meticulously acquired from TCGA, comprising a substantial collection of 521 cancerous tissue samples alongside 72 para-cancerous tissues, thereby providing a comprehensive overview of the tumor microenvironment. Additionally, datasets GSE213324 and GSE66271 were sourced from the GEO database, further enhancing the dataset for analysis. Furthermore, a total of 484 unique FRGs were curated from the FerrDb database (http://www.zhounan.org/ferrdb/legacy/index.html), which encompasses a diverse array of ferroptosis agonists, inhibitors, and marker genes, which were displayed in supplementary table S1.

### Functional analysis

Enrichment analyses, encompassing Gene Ontology (GO) and Kyoto Encyclopedia of Genes and Genomes (KEGG) assessments, were conducted on the differentially expressed genes identified from the intersection. The GO analyses comprised three categories: Biological Process (BP), Cellular Component (CC), and Molecular Function (MF). The GO terms and KEGG pathways that yielded P values less than 0.05 were deemed statistically significant, indicating a meaningful association and relevance in the biological context of the study.

### Screening crucial genes for the model

The most relevant model for prognosis was constructed by the LASSO-COX regression model. The risk score formula for each ccRCC patient were as follows.

$$\text{Risk score}= {\sum }_{i=1}^{n}\text{Coef}i\times Xi$$

Coef represents gene coefficients and X represents expression levels of genes.

The risk score median was used to divide the sample into high and low risk groups. The predictive prognostic ability of the model was assessed by KM curves and ROC. Univariate and multivariate COX were used to judge the model’s clinical relevance and assessed whether it could be used as an independent factor. Furthermore, nomogram had been constructed to predict patients’ survival rate at 1, 3, 5 years, with risk score as one of the prognostic factors, and survival calibration curves at 1, 3, 5 years had been plotted.

### Analysis of immune cell infiltration

The tumor purity, along with the abundance of immune and stromal cells, was meticulously assessed for patients classified into high- and low-risk groups using the advanced ESTIMATE package, which is specifically designed to provide insights into the tumor microenvironment. Furthermore, single-sample Gene Set Enrichment Analysis (ssGSEA) was employed to conduct a comprehensive evaluation of immune cell infiltration and to assess the activity levels of various immune-related functions in both patient cohorts. This approach offers a detailed understanding of the immune landscape within these distinct populations.

### Gene set enrichment analysis (GSEA)

GSEA is a computational method that determines whether an a priori defined set of genes shows statistically significant, concordant differences between two biological states [15]. Normalized enrichment scores (NES) were obtained by analyzing gene sets through 1000 permutations. A gene set was deemed significantly enriched if it exhibited a normal p-value of less than 0.05.

### Analysis of tumor mutations

Tumor mutation data were meticulously obtained from the comprehensive database of TCGA. Following this, the tumor mutation burden was calculated for each individual sample, facilitating a detailed analysis of its correlation with risk factors in both high-risk and low-risk groups. Subsequently, visually compelling waterfall plots were generated to effectively illustrate the distribution and significance of these mutations across different risk categories, thereby providing a clearer understanding of the underlying genetic variations associated with tumor progression.

### Statistical analysis

In this article, we employed the robust statistical software R version 4.2.0 for all facets of our data analysis and visualization, ensuring that our findings were both precise and visually engaging. The R packages utilized in our analysis encompassed a diverse range of tools, including ‘‘DEseq2’’ for differential expression analysis, ‘‘glmnet’’ for regularized regression, ‘‘survival’’ and ‘‘survminer’’ for survival analysis, ‘‘tROC’’ for receiver operating characteristic curve evaluation, ‘‘ClusterProfile’’ for functional enrichment analysis, and ‘‘ggplot2’’ for crafting elegant and informative visualizations. Furthermore, we leveraged ‘‘dplyr’’ for data manipulation, ‘‘maftools’’ to analyze mutation data, and ‘‘rms’’ to implement regression modeling strategies. We also relied on the base package “stats” for fundamental statistical functions while reiterating the use of “glmnet” due to its robust capabilities in managing high-dimensional datasets. Throughout our study, we established a threshold for statistical significance by considering results with a P-value of less than 0.05 as noteworthy indicators of meaningful relationships within the dataset.

## Result

### Identification and functional analysis of genes associated with ferroptosis in ccRCC

**Fig. 1:**
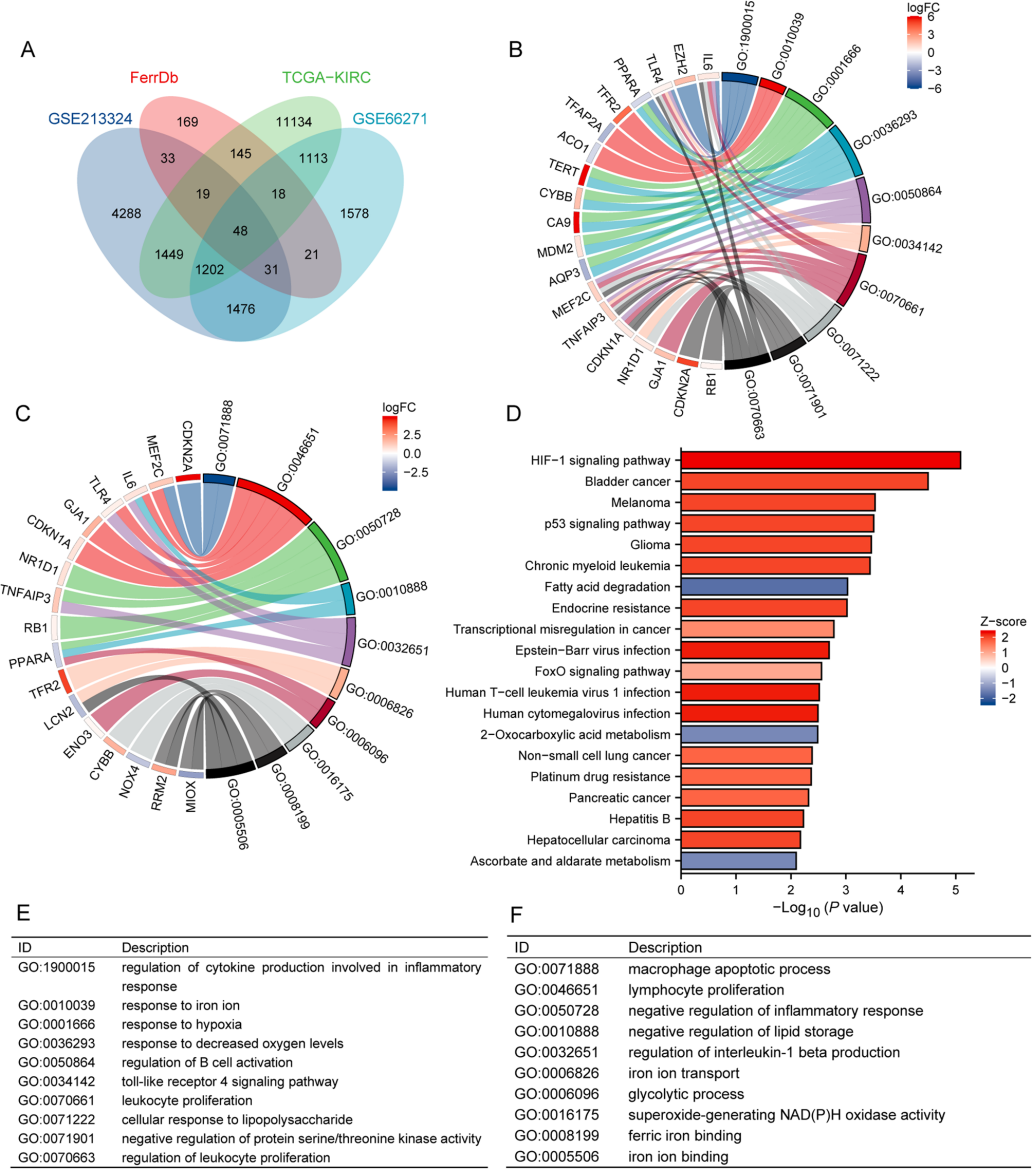
Screening for target genes. **A **Venn diagram showed the overlapping genes in the TCGA, GSE213324, GSE66271 and FerrDb. **B**–**C**, **E**–**F** GO analysis revealed biological functions for intersecting genes. **D** KEGG analysis showed the pathways for intersecting genes

The baseline characteristics of ccRCC patients from TCGA were listed in Table 1. To acquire prognostically relevant ferroptosis genes, univariate cox analysis was used to search prognostic-related genes in TCGA-KIRC, and then crossed with ferroptosis genes in the GSE213324, GSE66271 and FerrDb, which yielding 48 prognostic-related ferroptosis genes (Fig. 1A). Then, to explore their biological functions and pathways of these genes, we subjected these genes to GO and KEGG analyses in conjunction with LogFC (Fig. 1B–F). GO results demonstrated the role of these genes in regulating B cell activation, glycolytic processes, regulation of cytokine production involved in inflammatory responses (Biological Process) and NAD(P)H oxidase activity for superoxide production (Molecular Function). KEGG results showed that these genes were involved in the HIF-1 signaling pathway, ferroptosis and p53 signaling pathway.

**Table 1:** Correlation of clinical characteristics of patients in the high- and low-risk groups

Characteristics	Riskscore_low	Riskscore_high	P value
n	261	260	
Gender, n (%)			0.006
Male	155 (29.8%)	184 (35.3%)	
Female	106 (20.3%)	76 (14.6%)	
Age, median (IQR)	60 (51, 69)	61 (53, 70)	0.276
Pathologic.stage, n (%)			< 0.001
Stage III	41 (7.9%)	81 (15.5%)	
Stage I	175 (33.6%)	85 (16.3%)	
Stage IV	19 (3.6%)	64 (12.3%)	
Stage II	26 (5%)	30 (5.8%)	
Race, n (%)			0.774
White	226 (44%)	227 (44.2%)	
Black or african american	27 (5.3%)	26 (5.1%)	
Asian	5 (1%)	3 (0.6%)	
T.stage, n (%)			< 0.001
T3	53 (10.2%)	123 (23.6%)	
T1	178 (34.2%)	88 (16.9%)	
T2	29 (5.6%)	39 (7.5%)	
T4	1 (0.2%)	10 (1.9%)	
N.stage, n (%)			0.011
N1	2 (0.8%)	13 (5.1%)	
N0	112 (44.3%)	126 (49.8%)	
M.stage, n (%)			< 0.001
M0	227 (46.1%)	187 (38%)	
M1	18 (3.7%)	60 (12.2%)

### Construction of a prognostic model for FRGs

**Fig. 2:**
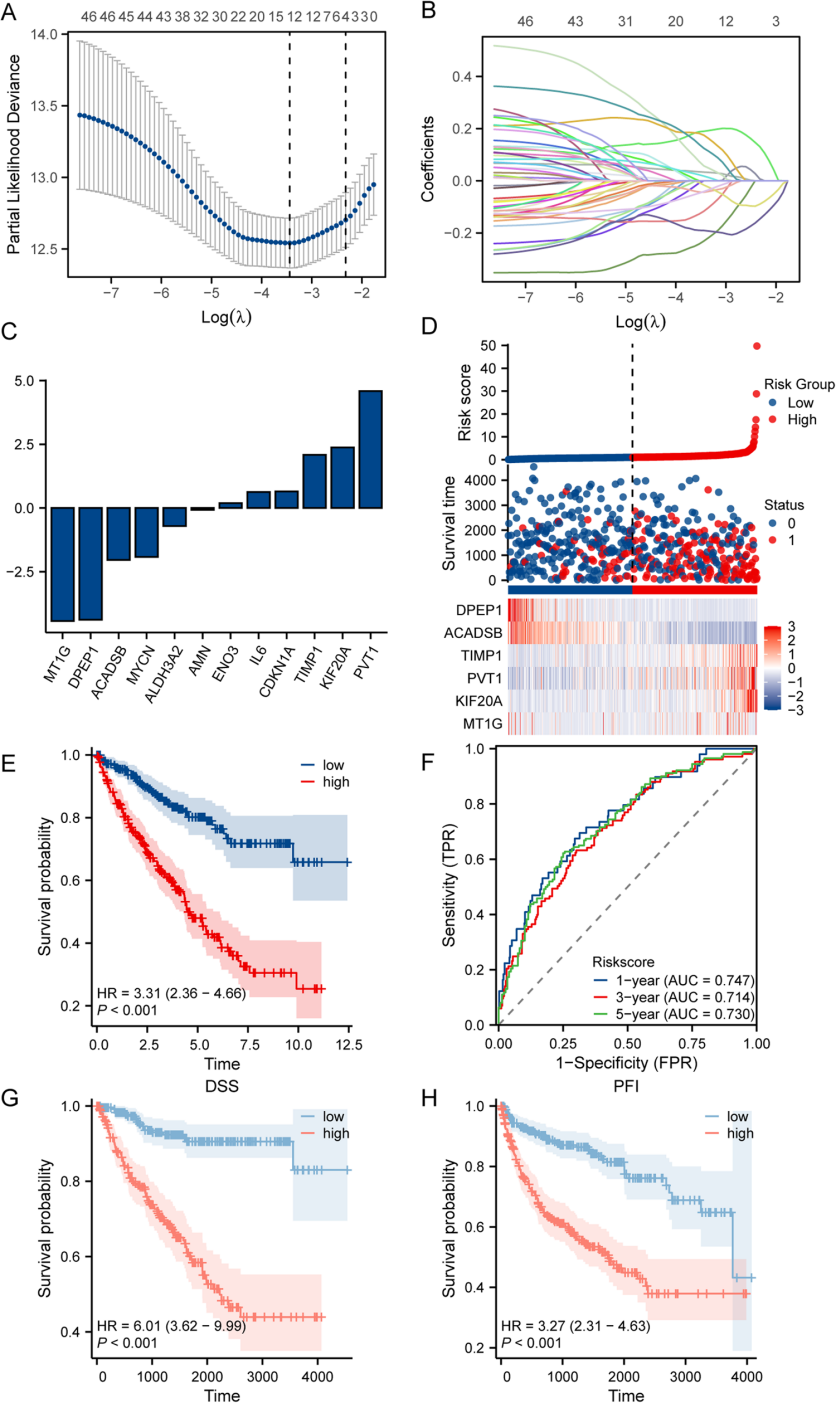
Construction of 6-ferroptosis-related marker genes and analysis of prognostic potential. **A**, **B** Least absolute shrinkage and selection operator (LASSO) analyzed the expression and survival information of genes. **C** Bar graph showed the expression of 12 genes. **D** The risk score, survival status, and expression of six FRGs in patients with ccRCC. **E** Kaplan–Meier curves showed that OS was significantly different between the high- and low-risk groups in ccRCC. **F** The signature was shown by the time-dependent ROC curve for predicting 1, 3, and 5 year survival. **G**–**H** Kaplan–Meier curves showed that DSS and PFI were significantly different between the high- and low-risk groups in ccRCC

To construct a prognostic risk model through these ferroptosis genes, LASSO regression analysis was used to identify 12 genes associated with ferroptosis and prognostically related to ferroptosis (Fig. 2A–C). Eight genes with absolute LogFC values greater than 1 were then selected for multivariate cox regression, which identified six genes involving in model construction, including *ADACSB*, *DPEP1*, *KIF20A*, *MT1G*, *PVT1* and *TIMP1*. The formula for the ferroptosis risk score was: risk score = (−2.134e−02 × ACADSB) + (−1.250e–02 × DPEP1) + (7.981e−04 × MT1G) + (0.02431 × KIF20A) + (0.1395 × PVT1) + (1.185e−04 × TIMP1). Then ccRCC patients were divided into high-risk and low-risk groups based on the median risk value. The distribution of risk scores, survival time and expression levels of six genes in different groups were showed in Fig. 2D. In the high-risk group, the mortality status was more intensive with high expression of *KIF20A*, *PVT1* and *TIMP1*, while low expression of *ADACSB*, *DPEP* and *MT1G*. The results of the KM survival curves suggested that ccRCC patients with high-risk scores had a significantly lower probability of survival than those with low-risk scores (Fig. 2E). And then, to assess the efficiency of the model, we created ROC curves. As shown in Fig. 2F, the areas under the curve (AUC) at 1, 3 and 5 years were 0.747, 0.713 and 0.730, respectively, supporting that the model has a beneficial diagnostic value for ccRCC. In addition, the results of the disease specific survival (DSS) and progression free interval (PFI) survival curves were analyzed, which suggested that ccRcc patients with high-risk scores had a significantly lower probability of survival than those with low-risk scores (Fig. 2G–H). Finally, the expression of these six genes in TCGA-KIRC was showed independently (Figure S1). These results suggested that the prognostic signature can effectively differentiate between high and low groups.

### Models could be used as independent prognostic indicators

**Fig. 3:**
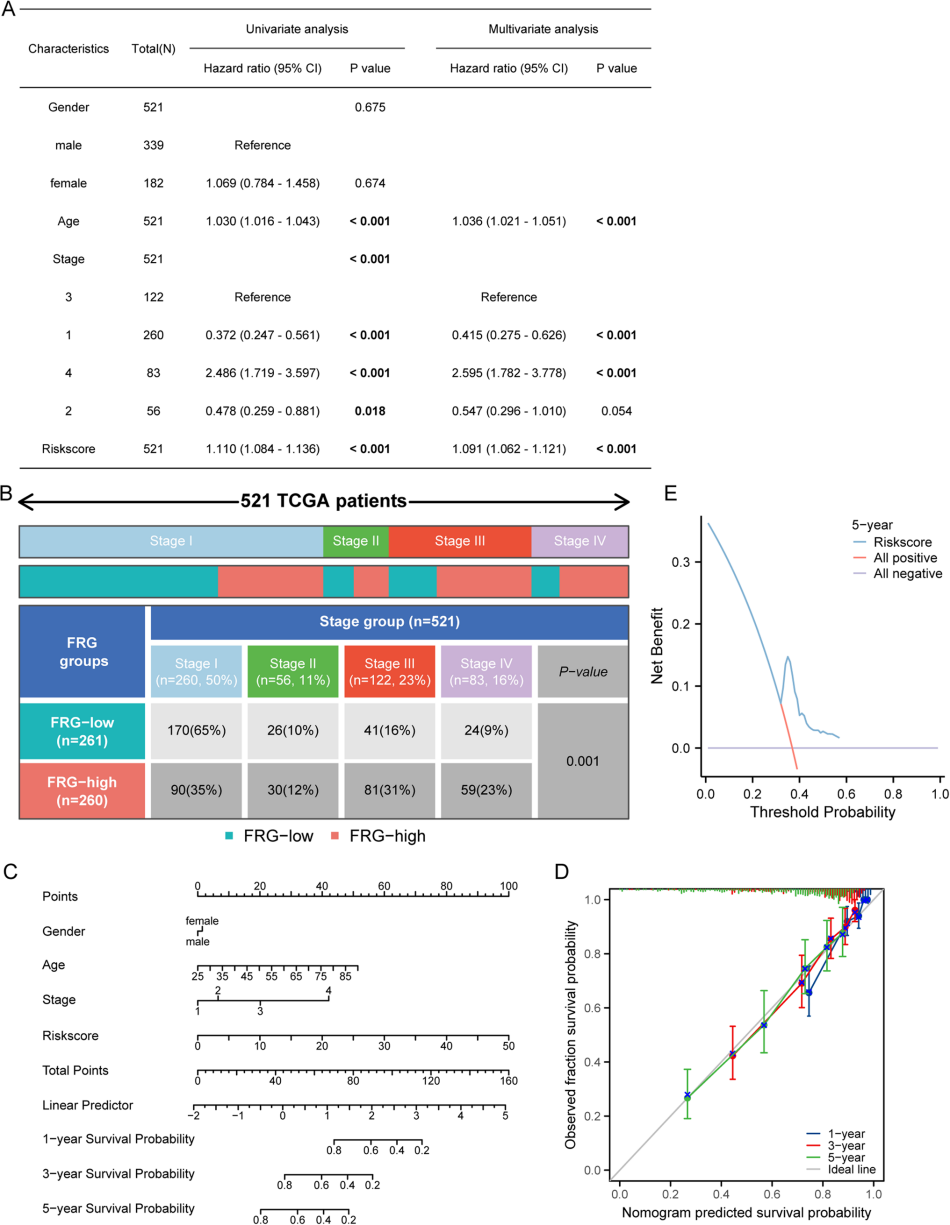
The analysis of independent prognosis. **A** Univariate Cox regression and multivariate Cox regression analysed of clinical characteristics and risk scores. **B **The difference in tumor staging between the high- and low-risk groups of patients. **C** 1-, 3-, and 5 year nomogram for predicting OS of ccRCC. There were three components in this nomogram: gender, age and stage. **D** The calibration curves were used to validate the nomogram for predicting 1-, 3- and 5- years survival of the patients. **E** The DCA showed the predictive of model

To further investigate the reliability of the prognostic models, we conducted an analysis to determine whether clinically relevant factors (such as gender, age, and stage) and risk scores could serve as independent prognostic indicators using univariate and multivariate COX regression. The risk score was significantly associated with overall survival (OS) as showed by the results of univariate COX regression (HR = 1.110, 95% CI  1.084–1.136, *P* < 0.001), and further multivariate COX regression indicated that the risk score could be used as an independent predictor for OS (HR = 1.091, 95% CI  1.062–1.121, P < 0.001) (Fig. 3A). Meanwhile, we performed a separately analysis of tumor staging and found significant differences in the staging of high- and low-risk patients, which could also be considered as an independent prognostic indicator for OS (Fig. 3A–B). The relationship between risk scores and clinical parameters were also further analyzed, which showing significant differences in patients across both high and low-risk groups when stratified by TNM stages (Figure S2). Furthermore, we constructed a nomogram to visually assess the prognosis of patients with tumors. To predict the survival probability of ccRCC patients, we used gender, age, stage, and risk score to establish a nomogram (Fig. 3C), which can be applied to predict the survival probability at 1, 3, and 5 years. In addition, the calibration curve of the nomogram showed that the actual survival rates at 1, 3, and 5 years were highly consistent with the predicted values (Fig. 3D), indicating that the model we constructed was reliable and accurate. For the further clinical evaluation of the FRG signature model, decision curve analysis (DCA) curve was further used to improve the predictive performance metrics of the 6-gene signature. As showed in Fig. 3E, the risk score of FRGs is correlated with better clinical outcomes, suggesting our 6-gene FRG signature can be better predictive model for prognostic prediction of ccRCC patients. Collectively, all these results demonstrated that the risk score of FRGs model show potential ability to predict the prognosis of ccRCC patients.

### Assessment of the ccRCC immune microenvironment

**Fig. 4:**
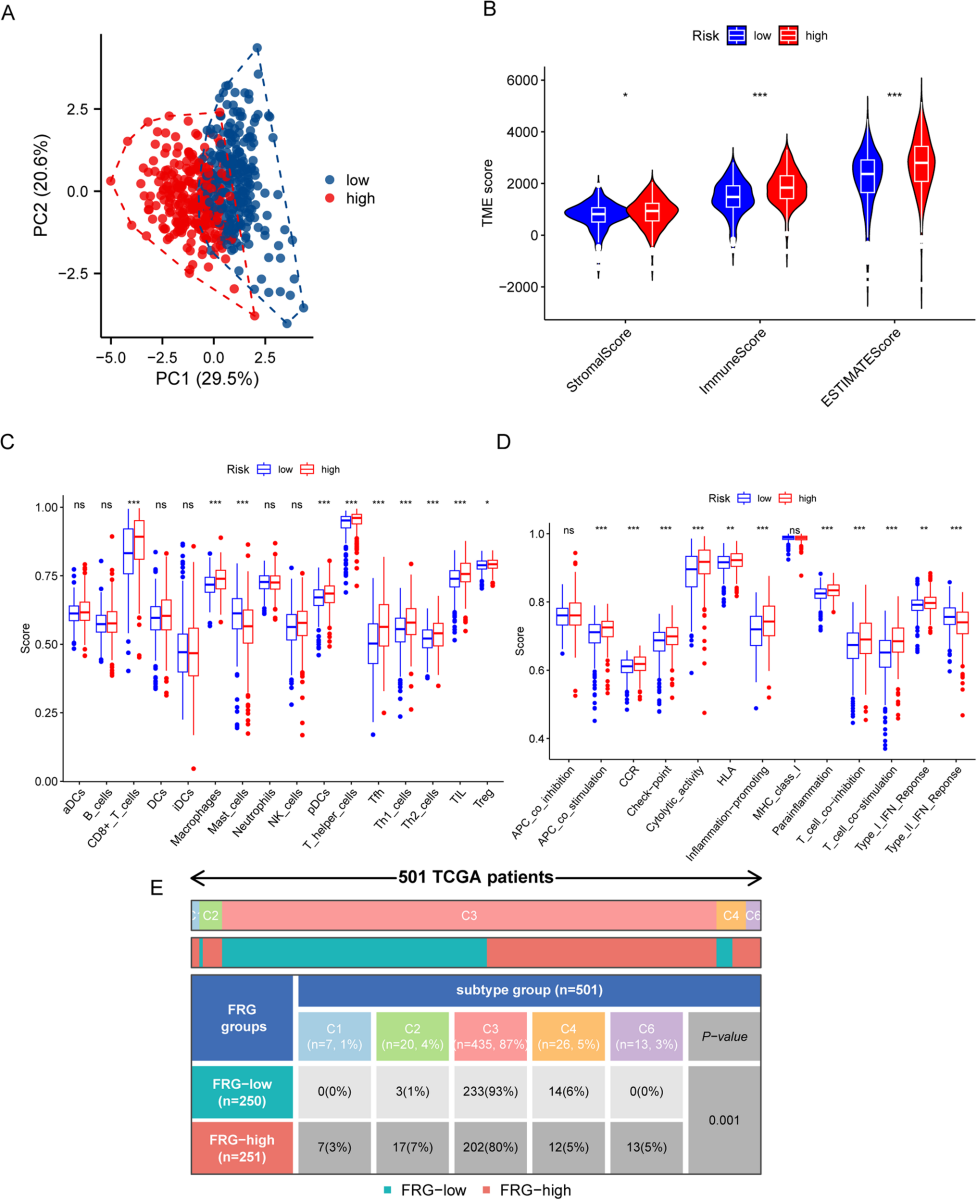
Immune microenvironment in high and low risk groups. **A** Principal component analysis plots. **B** Differences in immune-related scores between the high and low-risk groups. **C**, **D** The immune cells and function analysis based on ssGSEA. **E** Immunological subtypes in patients

Immune infiltration has particularly crucial roles in the development of tumors. Prior to conducting the analysis, we initially utilized principal component analysis (PCA) to downscale the high and low risk groups (Fig. 4A). For better immunological analysis, we first compared the differences in the tumor microenvironment between patients in the high- and low-risk groups and found that stromal score, immune cells and estimate score were higher in the high-risk group (Fig. 4B). Immune cells correlation results demonstrated significant differences in CD8 + T cells, macrophage, mast cells, plasmacytoid dendritic cell (pDC), T helper cells, T follicular helper cell (Tfh), T-helper 1 (Th1), T-helper 2 (Th2), tumor infiltrating lymphocyte (TIL), Treg cells in patients of high and low risk groups, and the infiltration of these immune cells was higher in the high-risk group (Fig. 4C). We then performed an immune function correlation analysis and found that antigen presenting cell (APC) co-stimulation, chemokine receptor (CCR), checkpoint, cytolytic activity, human leukocyte antigen (HLA), inflammation promoting, parainflammation, T-cell-co-inhibition, T-cell-co-stimulation and type I-IFN response were more relevant in the high-risk patient group (Fig. 4D). All the markers of immune function and immune cells mentioned in supplementary table S2 are included. To determine whether there was a difference in the immunophenotyping of patients in the high- and low-risk groups, we grouped the patients into C1-C6 (C1 (wound healing), C2 (IFN-γ dominant), C3 (inflammatory), C4 (lymphocyte depleted), C5 (immunologically quiet), C6 (TGF-β dominant)) groups and discovered that 501 patients had a specific phenotype, and we also observed a significantly different phenotype between patients in the high- and low-risk groups (Fig. 4E). The above results suggested that there were differences in the immune microenvironment between patients in the high and low risk groups, which provided a reference for the clinical use of immunotherapy. These results also demonstrate the potential roles of the constructed prognostic model in ccRCC immunotherapy.

### The FRGs model is relevant to ICI treatment

**Fig. 5:**
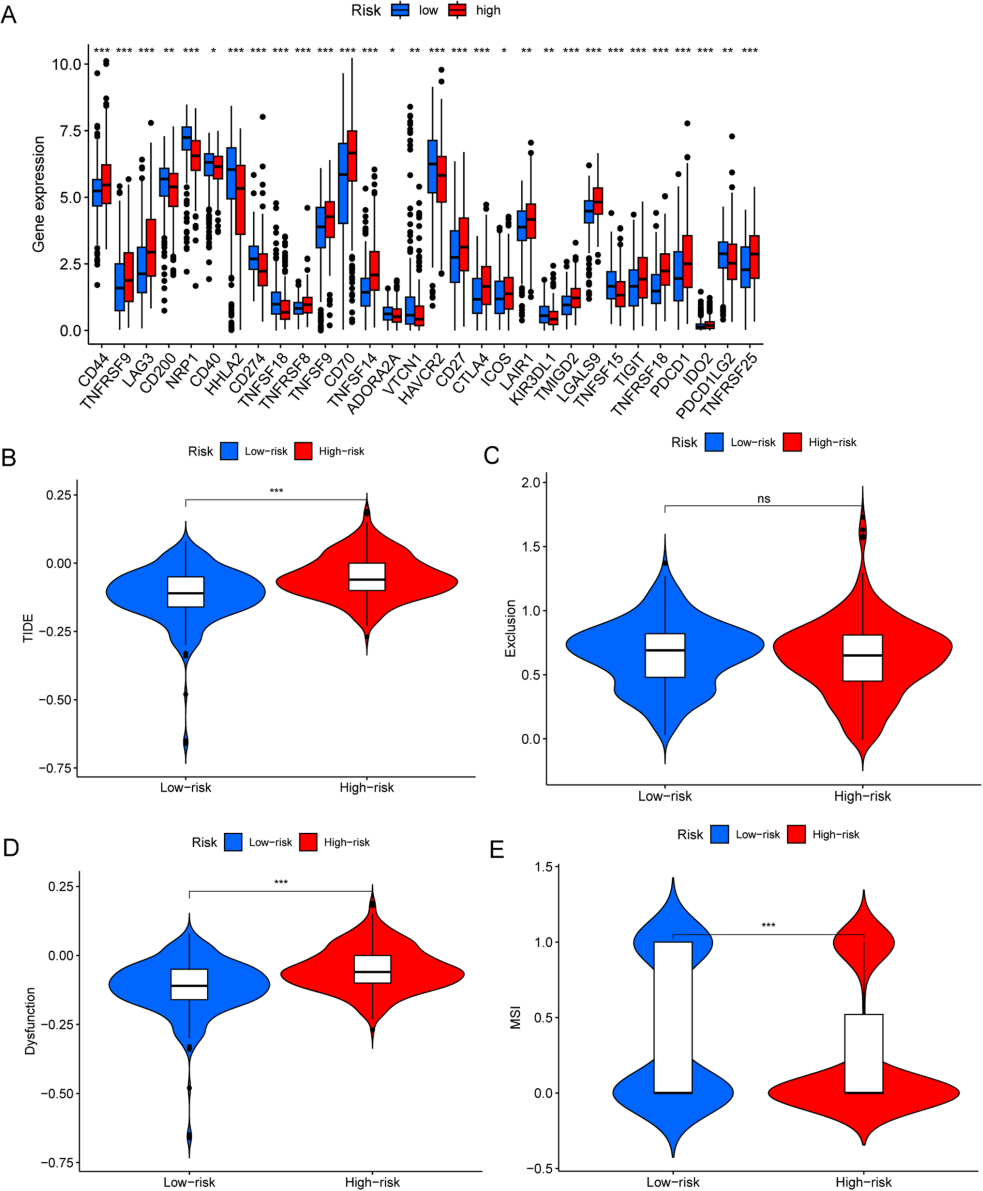
The immune cells and subtypes of the FRGs risk group. **A** Differential expression of immune checkpoint-related genes in high- and low-risk groups. **B**–**E** TIDE, exclusion, dysfusion and MSI score between high- and low-risk group

Given the important role of ICI in ccRCC treatment, we further analyzed the differential expression of immune checkpoint genes between the high-risk and low-risk groups. As shown in Fig. 5A, patients in the high-risk group had higher expression of immune checkpoint genes, such as lymphocyte activation gene-3 (LAG3), programmed cell death 1 (PDCD1) and cytotoxic T lymphocyte-associated antigen-4 (CTL4), compared to the low-risk group. The TIDE score and exclusion score were significantly higher in the high-risk group, with no significant difference in the dysfunction score and lower microsatellite instability (MSI) score, suggesting that the patients in the high-risk group had increased immune escape potential and that the efficacy of ICI may be poorer (Fig. 5B–E). These results suggest that the prognostic characteristics of the two groups are distinguished ICI treatment differences.

### Discovery of pathway enrichment in high and low risk groups analyzed by GSEA

**Fig. 6:**
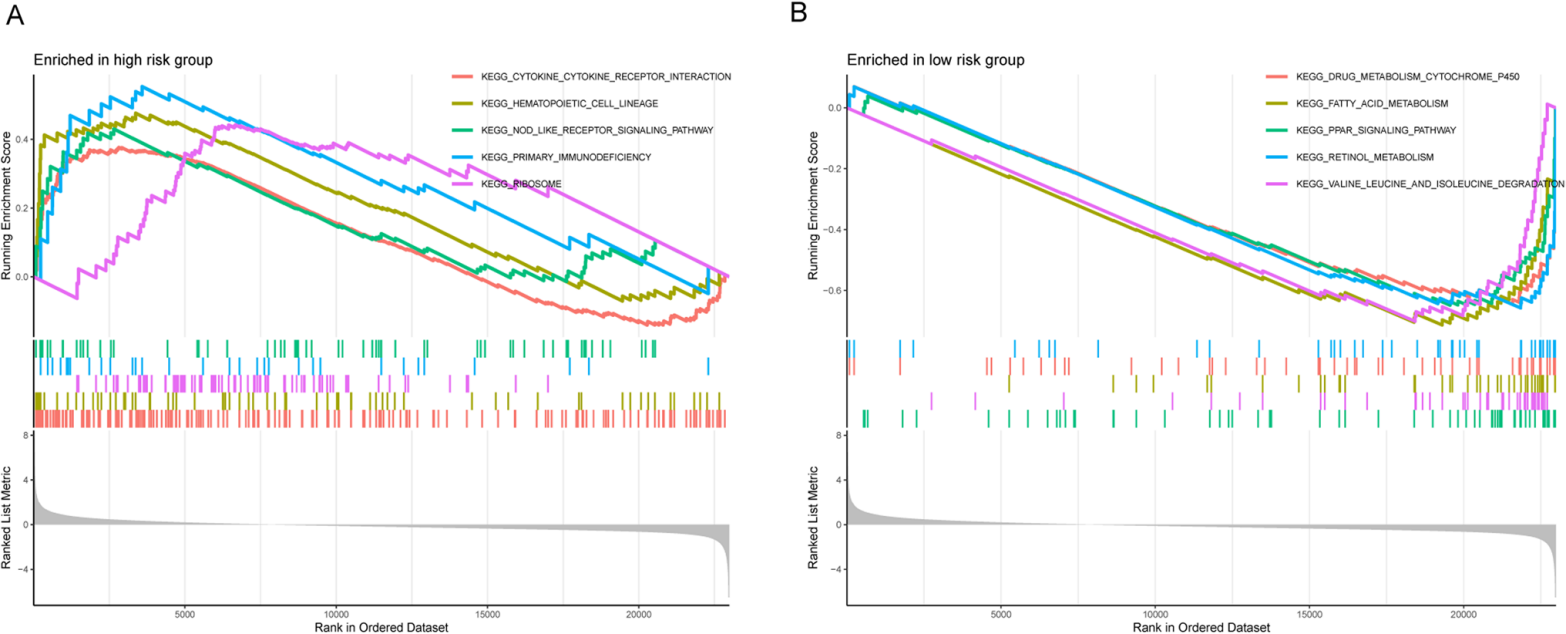
Pathway enrichment analyses were performed for high- and low-risk groups based on FRGs prognostic characteristics. **A** GSEA showed a significant enrichment of the top 5 pathways in patients at high risk for ccRCC. **B** GSEA showed a significant enrichment of the top 5 pathways in patients at low risk for ccRCC

To explore potential differences in signaling pathways between different risk groups classified by FRGs characteristics. We determined the DEG between high and low risk groups by setting cut-off values of log2|FC|> 1 and FDR < 0.25, and then analyzed them using GSEA (*p* < 0.05). The results revealed that high risk was mainly enriched in hematopoietic cell lineage, primary immunodeficiency, Nod-like receptor signaling pathway (Fig. 6A). While the low-risk group was mainly enriched in metabolism-related pathways, such as fatty acid metabolism, retinol metabolism, drug metabolism (Fig. 6B). The detailed information on the results of the GSEA analysis was listed in supplementary table S3.

### The patients with high-risk scores present high TMB

**Fig. 7:**
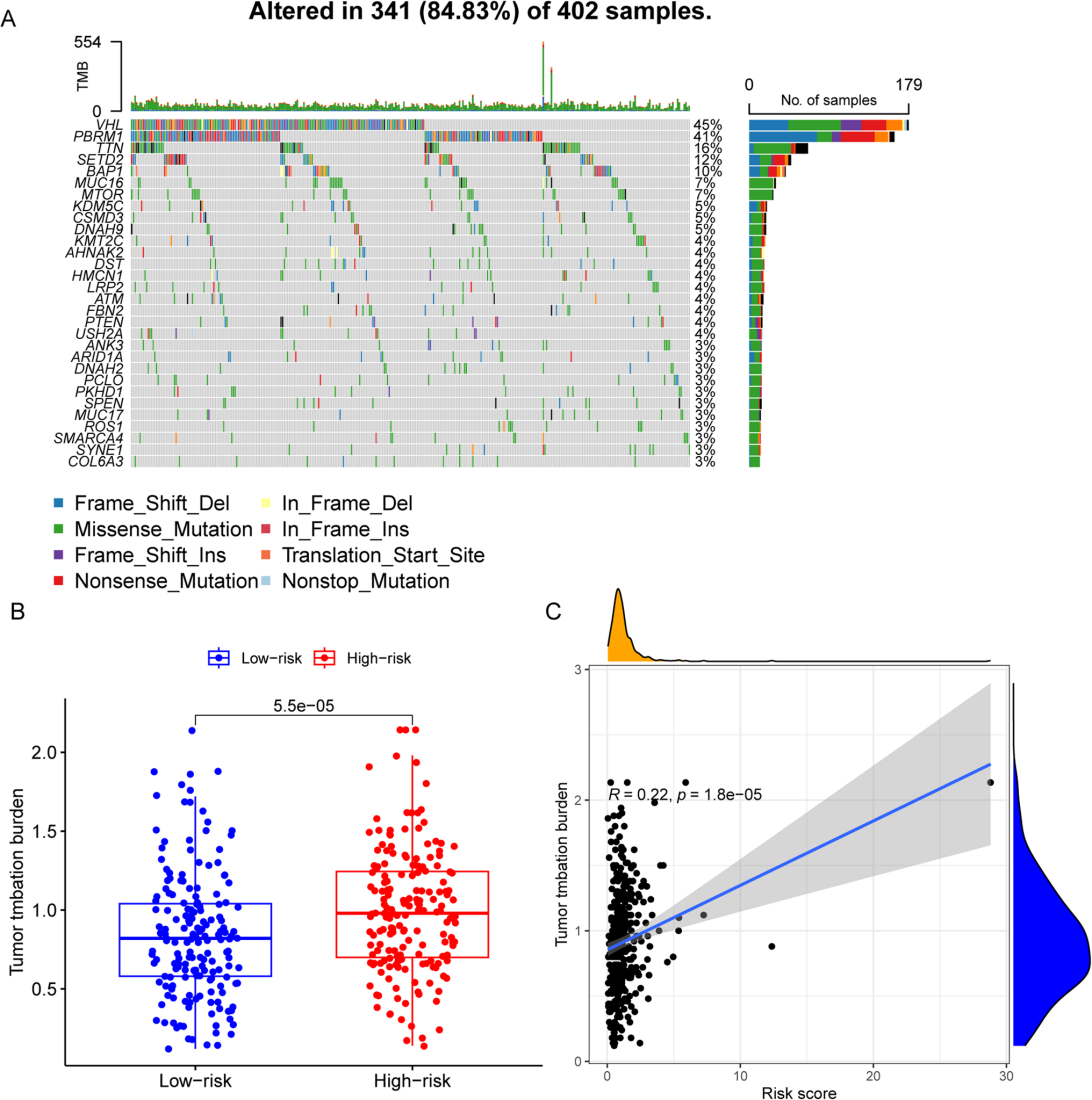
The FRGs risk score and groups correlated with TMB. **A** Genes with top 30 mutation frequencies. **B** The TMB of patients in the high FRGs risk group (red points) was higher than those in the low FRGs group (blue points). **C** The patients’ TMB positively correlated with FRGs risk score

We extracted patients' mutation information and showed the top 30 genes in terms of mutation frequency (Fig. 7A). Through the TMB analysis, we noticed that there was a significantly different TMB between patients in the high- and low-risk groups and that patients in the high-risk group had a higher tumor mutation load (Fig. 7B), while the risk score was positively correlated with the TMB (Fig. 7C).

## Discussion

CcRCC is one of the most aggressive types of malignant renal cancer in which patients usually have a poor prognosis [9, 16]. Although surgical resection is the mainstay of clinical treatment for ccRCC, metastases are found in some patients at the early stage of diagnosis, and metastases are found in 40% of patients during follow-up, even with positive surgical treatment [17]. In addition, renal cell carcinoma is one of the most immune-infiltrated tumors [18–20].The molecular mechanism underlying its development has not yet been fully elucidated, which hinders early diagnosis and treatment. Therefore, it is of great significance to explore ccRCC biomarkers and potential therapeutic targets. Iron is a fundamental trace element in the human body, primarily involved in the synthesis of hemoglobin, myoglobin, and other important substances, as well as playing a vital role in numerous biological functions. However, an overload or deficiency of iron can lead to cell death and disrupt the normal functions of the organism. Ferroptosis is a novel form of cell death that was discovered in 2012 [6]. It is now also demonstrated in a large number of studies that iron plays a role in many different tumor tissues, such as HCC [21], stomach adenocarcinoma (STAD) [9], and triple-negative breast cancer (TNBC) [22]. Consequently, FRGs can be used as potential markers for predicting tumor progression. Moreover, tumor markers that are simultaneously associated with both ferroptosis and prognosis would be of great significance for the diagnosis and treatment of ccRCC patients.

In this study, we conducted a comprehensive analysis of data from TCGA, GEO, and FerrDb and successfully identified differentially expressed FRGs that consistently correlated with both prognosis and ferroptosis. Subsequent analyses were performed using the constructed model, including prognostic analysis, tumor immune infiltration analysis, and tumor mutation analysis, all of which demonstrated the excellent performance of the model. A promising prognosis is certainly encouraging for the patient. Through risk scoring, patients were divided into high and low-risk groups, with survival analysis clearly indicating poorer prognosis for those in the high-risk group and better prognosis for those in the low-risk group. Our model had a clearer and more meaningful distinction between patients in the high and low risk groups, when compared with endoplasmic reticulum stress-related gene signature model [23]. We also observed that both DSS and PFI were associated with poorer survival outcomes in patients classified within the high-risk group. This suggested that individuals exhibiting high expression levels of these genes are more challenging to treat effectively in a clinical setting. Conversely, patients belonging to the low-risk group are likely to experience an improved cure rate. Following this, ROC analysis was performed, and the results displayed that the FRGs model had a satisfactory predictive ability and the AUC value of the model could reach a maximum of 0.747. Compared to the model constructed with cuproptosis-related genes used by Liu et al., the AUC value of our FRGs model was 0.08, which was higher than the AUC value of their model [24]. Meanwhile, the AUC value was 0.102, which was higher compared to the redox-related genes used by Ren et al. [25]. Although our AUC values were slightly higher than aging and senescence-related gene signature model, the overall survival of patients in the high and low risk groups of our model slightly poor [26]. We have conducted a composite ability calculation on the model, yielding an excellent result. It is noteworthy that our model demonstrates a high level of independence, indicating its potential for reasonably accurate prediction of patient prognosis. Furthermore, we have utilized the nomogram model to analyze patient prognosis based on various factors, and the calibration curves indicate that our model accurately predicts the incidence of OS at 1, 3, and 5 years compared to the ideal predictive model. Our model was expected to provide greater relevance and significance in assessing OS when compared with ferroptosis-related signatures model [27]. The joint application of multiple models may provide better prediction results. Meanwhile, the DCA curve showed a good clinical benefit. In summary, our FRGs model enables fairly accurate prediction of patient prognosis and may prove useful in informing individualized patient treatment while also potentially reducing doctors' workloads in certain cases. Small molecule inhibitors may be developed based on model genes to specifically target the relevant genes involved in regulating the progression of kidney cancer in the future.

It has been previously shown that ferroptosis is intimately related with tumor immunity and may even have an impact on the function of immune cells. Therefore, modulation of ferroptosis may be an opportunity to influencing patient immunotherapy. Meanwhile, features of the tumor microenvironment heavily affect disease biology and may affect responses to systemic therapy [28, 29]. For the immune status of ccRCC patients, our tumor purity analyses revealed significantly higher immune, stromal, and estimate scores in the high-risk group compared to the low-risk group. Additionally, we conducted ssGSEA analyses of 13 immune-related pathways and 16 immune cell subsets and discovered that patients in the high-risk group almost always with higher scores, although there were also fractions that were not significantly different between the two groups or were higher in the low-risk group. According to the analysis, it is easy to identify that the IFN-I(α/β) response may serve as one of the breakthroughs in the treatment of ccRCC. The high-risk group has more aggregation of pDC, while the ability of pDC to produce IFN-I(α/β) is dependent on the interferon response family (IRF), including IRF8 [30] and IRF5 [31]. Therefore, a series of inflammatory responses induced by IFN-I(α/β) could be diminished by restricting the expression of IRF family. On the one hand, there are more Th1 cells in high-risk groups, which is mainly generated by IFN-γ-induced differentiation, in which IFN-γ will activate downstream signal transducer and activator of transcription 1(STAT1) to generate cytokine storm [32, 33]. Therefore, patients may be benefited by reducing IFN-γ production, inhibiting STAT1 transcription and IFN-γ receptor on the surface of Th1 to alleviate the disease. Meanwhile, inhibition of the STAT4 transcription may also be the therapeutic key as IL-12 can potentially activate STAT4 to induce IFN-γ production and further stimulate Th1 differentiation [34, 35]. Subtyping analysis of tumor immunity revealed that ccRCC patients were mainly clustered in three subtypes, C2 (IFN-γ dominant), C3 (inflammatory) and C4 (lymphocyte depleted) [36], which is highly consistent with our previous suggestion that attenuating the inflammatory response might be able to therapeutically treat the tumor. The tumor immune checkpoint, such as PD-L1 and CTLA-4, are currently the most extensively and well-researched clinical studies [37–39]. The levels of these checkpoints were higher in the high-risk group compared to the low-risk group, suggesting that targeting them may be beneficial for patients seeking to regain their health. It is already known that IRF1 can induce PD-L1 expression, and the IFN-γ/IFNGR/JAK/STAT axis can modulate IRF1 [40]. Therefore, interfering with any of the targets on the IFN-γ/IFNGR/JAK/STAT/IRF1 axis will impact PD-L1 expression and ultimately affect resistance to the tumor. In order to assess the immunotherapy effect, our calculation based on the TIDE algorithm [41] showed that the scores of the high-risk group are higher than those of the low-risk group, from which it is determined that the patients in the high-risk group will be less efficient in responding to ICI therapy. CcRCC is essentially a metabolic disease characterized by a reprogramming of energetic metabolism [19]. In particular the metabolic flux through glycolysis is partitioned, and mitochondrial bioenergetics and OxPhox are impaired, as well as lipid metabolism [18, 42, 43]. The results of GSEA enrichment suggested that specific pathway receptors or cytokine receptors could be targeted to intervene in the high-risk group of patients, while critical targets of metabolism should be targeted in the low-risk group. These findings may serve as a valuable reference for immunotherapy in ccRCC patients.

This study also has some limitations. Firstly, the entire study was conducted using data mining techniques; however, the absence of independent validation through wet lab experiments involving cell lines or clinical specimens significantly undermines the robustness of this research. Therefore, at a later stage, we are also prepared to collect more clinical data to fully validate the reliability of the model, which partially requires a lot of time and money. Secondly, we have yet to understand the specific mechanism of action and function of the six-FRGs in ccRCC, and a large number of basic experiments are still needed for subsequent investigation.

## Conclusions

A novel risk scoring model, based on six FRGs (*ADACSB*, *DPEP1*, *KIF20A*, *MT1G*, *PVT1* and *TIMP1*), has been developed. This model has demonstrated the ability to predict survival outcomes. Additionally, a preliminary establishment of the relationship between the risk model and the immune environment has been made, which may aid in understanding the potential use of immunotherapy in cancer patients and facilitate the provision of clinical therapy treatment.

## Supplementary Information

Additional file 1

Additional file 2

Additional file 3

Additional file 4

## Data Availability

The data that support the findings of this study are openly available in The Cancer Genome Atlas Program (TCGA) (https://www.cancer.gov/ccg/research/genome-sequencing/tcga), ferroptosis database (FerrDB) (http://www.zhounan.org/ferrdb/current/), and Gene Expression Omnibus (GEO) database (https://www.ncbi.nlm.nih.gov/geo/). All data supporting the findings of this study were available from the first author or corresponding author upon reasonable request.
