# Supplementary material for: Identification of ferroptosis-related gene signatures as a novel prognostic model for clear cell renal cell carcinoma
Source: Discov Oncol. 2025 Apr 3;16:456. doi: 10.1007/s12672-025-02202-1 (PMC11968612; doi:10.1007/s12672-025-02202-1)
Supplement: Supplementary file 1 — Additional file 1 [file 12672_2025_2202_MOESM1_ESM.docx]

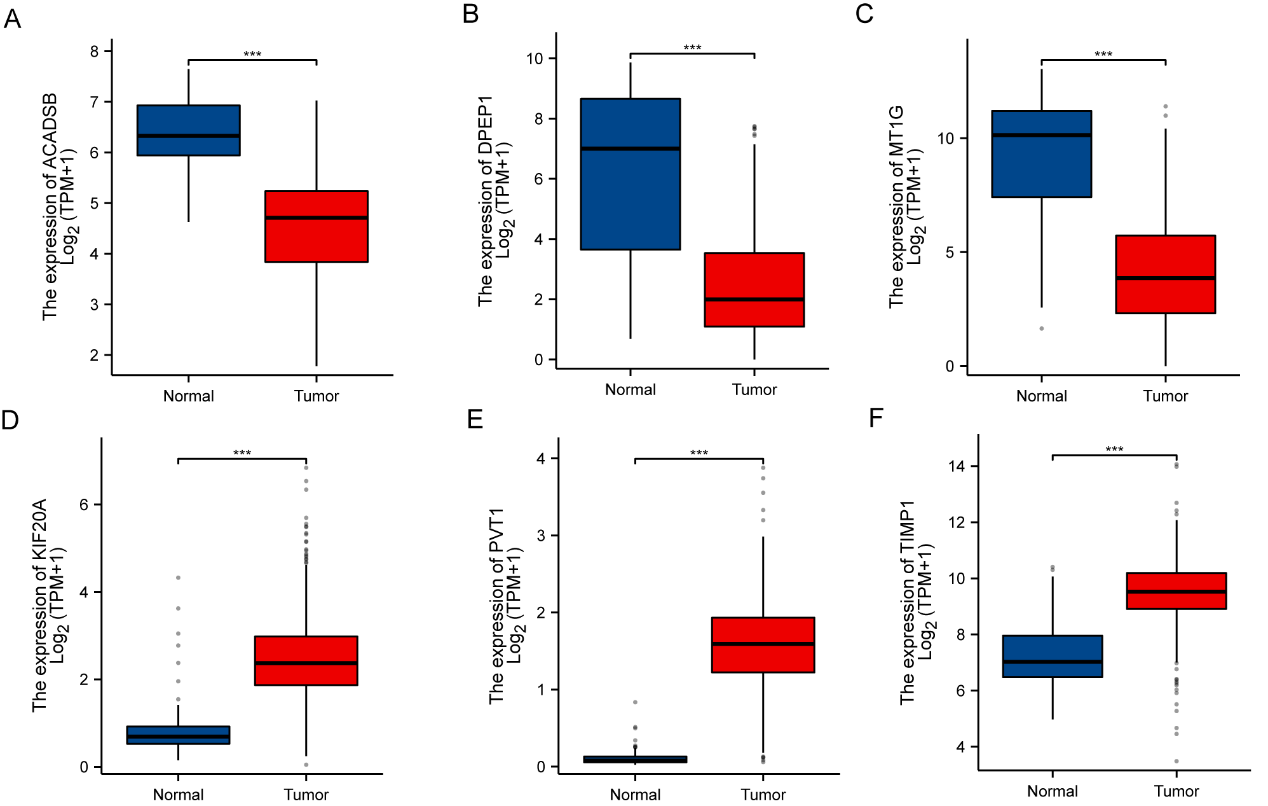


**Figure. S1** The expression of six FRGs in ccRcc. (A-C) The expression of *ACADSB*, *DPEP1*, *MT1G* were lower in tumor tissue. (D-F) The expression of *KIF20A*, *PVT1*, *TIMP1* were higher in tumor tissue.


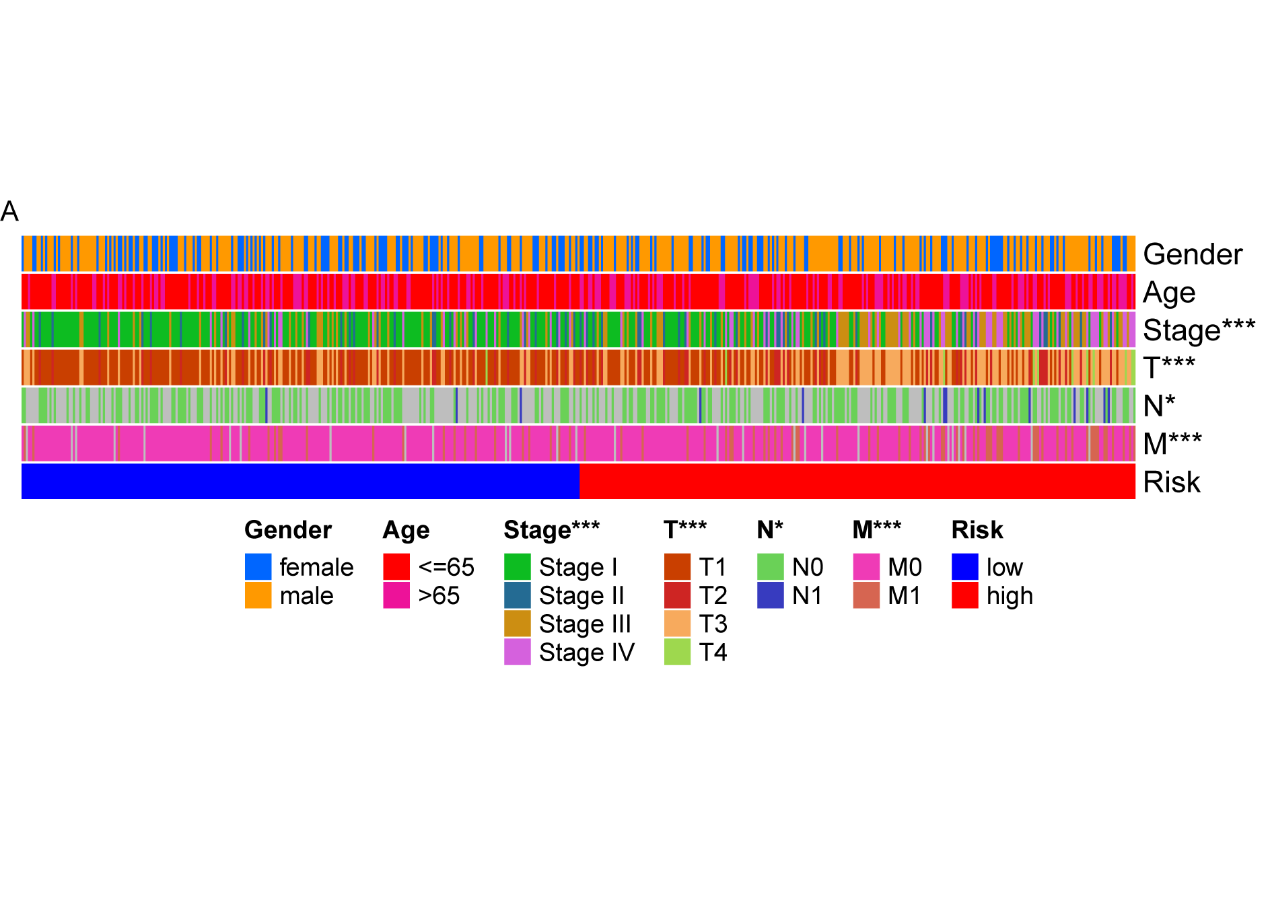


**Figure. S2** Relationship between risk scores and clinical factors.
